# Comparison of the mechanical properties and anchoring performance of polyvinylidene fluoride and polypropylene barbed sutures for tendon repair

**DOI:** 10.1002/jbm.b.35074

**Published:** 2022-06-08

**Authors:** Yihan Huang, Edwin R. Cadet, Martin W. King, Jacqueline H. Cole

**Affiliations:** ^1^ Wilson College of Textiles North Carolina State University Raleigh North Carolina USA; ^2^ Raleigh Orthopaedic Clinic Raleigh North Carolina USA; ^3^ Department of Orthopaedics University of North Carolina at Chapel Hill Chapel Hill North Carolina USA; ^4^ College of Textiles Donghua University Shanghai China; ^5^ Joint Department of Biomedical Engineering University of North Carolina at Chapel Hill and North Carolina State University Raleigh North Carolina USA

**Keywords:** barbed sutures, biomechanics, ex vivo, polypropylene, polyvinylidene fluoride, tendon repair

## Abstract

Polyvinylidene fluoride (PVDF) has been considered as an alternative suture material to replace polypropylene (PP) due to its superior biocompatibility and mechanical properties, but it has never been examined for use in barbed sutures, particularly for tendon repair. This study fabricated size 2–0 PVDF and PP bidirectional barbed sutures and compared their mechanical properties and anchoring performance in patellar tendons. The mechanical properties were evaluated via tensile testing, and the anchoring performance of the barbed sutures was assessed by a tendon suture pullout test. Sixty porcine patellar tendons were harvested, transected to mimic a full‐thickness injury, and repaired using a cross‐locked cruciate suturing technique. The ultimate tensile force was 60% higher for the PVDF barbed sutures (22.4 ± 2.1 N) than for the PP barbed sutures (14.0 ± 1.7 N). The maximum pullout force was 35% higher for PVDF barbed sutures (70.8 ± 7.8 N) than for PP barbed sutures (52.4 ± 5.8 N). The force needed to form a 2‐mm gap, indicative of repair failure, was similar between the PVDF (29.2 ± 5.0 N) and PP (25.6 ± 3.1 N) barbed sutures, but both were greater than the 2‐mm‐gap forces for non‐barbed sutures of the same size. In this study, PVDF barbed sutures provided better mechanical properties and improved tissue anchoring performance compared to the barbed PP sutures for porcine patellar tendon repair, demonstrating that PVDF monofilament sutures can be barbed and used effectively for tendon repair.

## 1 INTRODUCTION

As tough bands of fibrous connective tissue that connect muscle to bone, tendons regulate and control the forces between muscles and bones during joint movements. With tendon tears or ruptures, patients may need surgical repair or tenorrhaphy to prevent permanent functional deficits or impairment.^1^ The ideal tenorrhaphy should meet the following requirements: (1) ease of suture placement, (2) secure knots, (3) smooth end‐to‐end tendon apposition, (4) minimal gap or no gap at the repair site, (5) avoidance of injury to the tendon vasculature, and (6) sufficient strength for early active postoperative motions.^2^ Conventional surgical sutures require surgeons to tie secure knots at the end of the repair, which can be challenging, especially when space is limited. In addition, knots are potentially the weakest points, reducing the maximum holding capacity of the suture and widening the cross‐sectional area of the tendon.^3^

Barbed knotless sutures have recently gained more attention because they have certain advantages over traditional knotted sutures.^4^ With multiple barbs projecting from their surface and pointing parallel to each other in the direction away from the needle, barbed sutures can be passed through the tissue when pulled from the needle end and resist removal when pulled in the opposite direction. Since the barbs grasp the surrounding tissues, knots are unnecessary, making suturing during surgery both easier and faster. Without the presence of the knots, barbs distribute the anchoring stress along the length of the suture, applying more consistent tension across the wound and reducing the repair‐site cross‐sectional area, which facilitates the healing process.

In 1967, McKenzie first proposed the idea of using an internal multiple barbed suture to repair flexor tendons in a canine model, but due to poorly constructed barb configurations and unsatisfactory biomaterials, interest in using barbed sutures soon waned.^5^, ^6^ With recent developments in technology and biomaterials, a resurgence of interest has occurred, and the US Food and Drug Administration (FDA) has approved several commercial barbed nylon, polydioxanone (PDS), and polypropylene (PP) sutures over the last decade. Barbed sutures have attracted more attention and have been introduced into the surgeon's armamentarium for their specific advantages over traditional knotted sutures. In particular, they are being widely used in cosmetic and plastic surgeries, and also in other types of surgery that are space‐limited, such as in laparoscopic surgery and less invasive obstetric and gynecological surgeries.^7^, ^8^, ^9^ Ex vivo studies have investigated using size 2–0 and 3–0 PP barbed sutures for flexor digitorum profundus tendon repair in pigs and cadavers, and barbed sutures made from different materials were also used to evaluate their possible use for tendon repair in several animal and human models.^10^, ^11^, ^12^, ^13^, ^14^, ^15^, ^16^, ^17^ Two in vivo animal studies have examined the use of barbed sutures for tendon repair, one in 2015 using 3–0 Quill SRS PDS bidirectional barbed sutures in chickens to repair the flexor digitorum profundus tendon, and one in 2019 using 2–0 PP bidirectional barbed sutures in a canine case study to repair a complete common calcanean tendon rupture.^18^, ^19^ However, barbed sutures have not yet been used for human clinical tendon repair.

Although PP barbed sutures have been used widely for clinical skin closure and in plastic and general surgery, some reports have expressed dissatisfaction with PP monofilament sutures because of their thrombogenicity and reports of mechanical failure.^20^ Polyvinylidene fluoride (PVDF) has been considered as an alternative suture material to replace PP, because several ex vivo and in vivo evaluations have reported superior biomechanical performance, improved creep resistance, and greater biocompatibility and biostability of PVDF sutures.^8^, ^9^, ^21^, ^22^ For example, when PVDF and PP sutures were exposed to hydrolytic conditions for 9 years, the PVDF sutures lost only 7.5% of their initial tensile strength compared to 46.6% lost for PP sutures; and in a 2‐year in vivo study of a canine thoracoabdominal bypass model, surface stress cracking was visible on PP sutures but not on PVDF sutures, indicating more long‐term biostable potential for PVDF than PP.^8^, ^9^ The overall goal of this study was to evaluate and compare the performance of PVDF and PP knotless barbed sutures, in particular the anchoring performance, in the repair of tendon tissue. We hypothesized that PVDF barbed sutures will have superior mechanical properties and tissue anchoring for a full‐thickness tendon repair.

## 2 MATERIALS AND METHODS

### 2.1 Materials

Suture sizes 2–0 and 3–0 are commonly used for human flexor tendon repairs, and these sizes of barbed sutures were used in previous cadaver and animal in vivo repair studies.^11^, ^18^, ^19^, ^23^, ^24^, ^25^ Manufacturers report that the mechanical properties of barbed sutures are similar to those of non‐barbed sutures of one size smaller.^26^ Therefore, size 2–0 (non‐barbed and barbed) and 3–0 (barbed) sutures were examined in this study. PVDF and PP surgical sutures were obtained from G. Krahmer GmbH (Buchholz, Germany).

**FIGURE 1:**
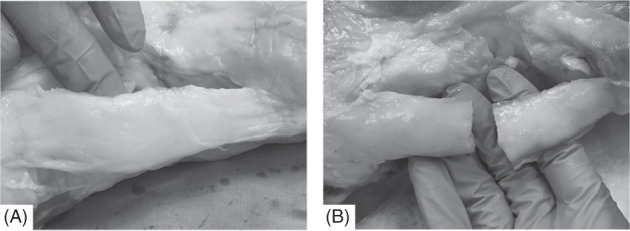
Patellar tendon (A) before and (B) after transection

Sixty porcine knees (30 pairs) from 3‐ to 4‐year‐old female pigs with an average weight of 561 lbs (423–716 lbs) were obtained from City Packing Company through Neese Country Sausage, Inc. (Burlington, NC). The knees were dissected to expose the patellar tendons, which were transected in the middle to mimic a full‐thickness injury (Figure 1) and then stored at −7°C until surgical repair and testing. Each tendon was randomly assigned to one of six different groups based on suture type (*n =* 8 each): 2–0 non‐barbed, 2–0 barbed, and 3–0 barbed sutures, each made from both PVDF and PP suture materials.

### 2.2 Barbed suture fabrication

**FIGURE 2:**
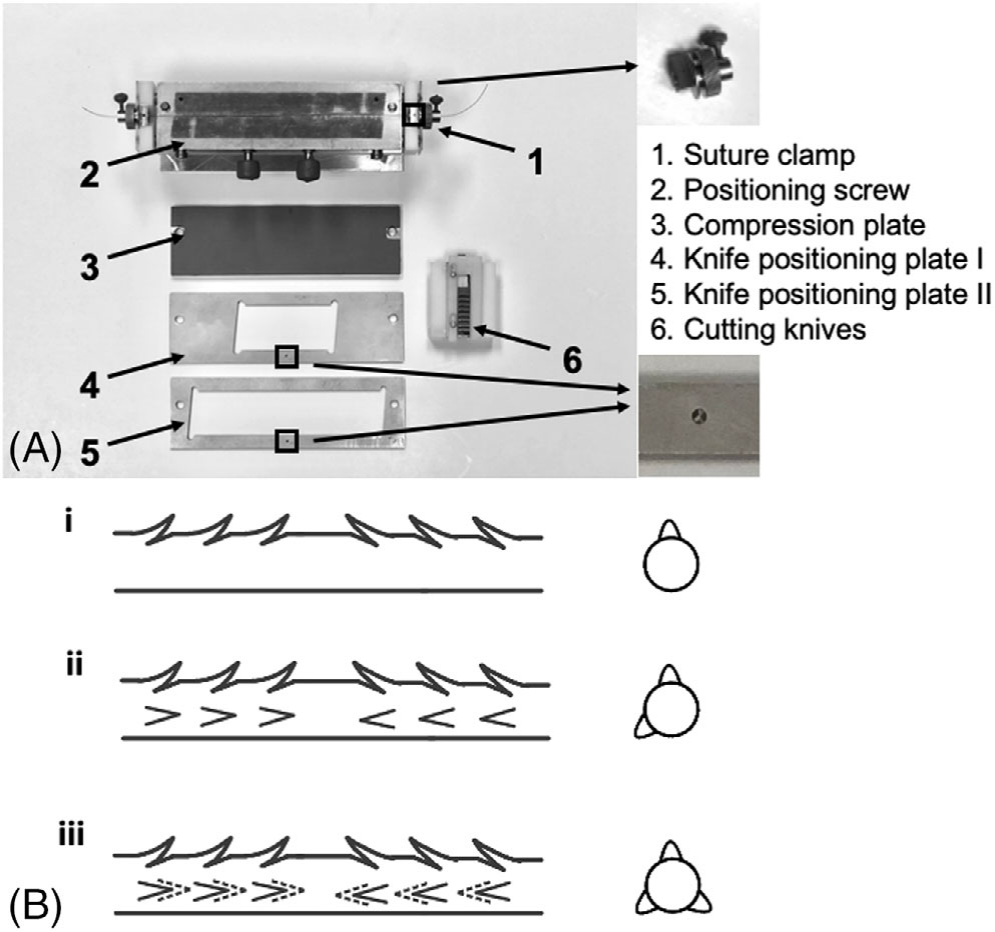
(A) Barb cutting machine with inset showing one dot on the knife positioning plate; (B) schematic of front view and side view of bidirectional barbed sutures after cuts were made using plates with (i) one dot; (ii) one and two dots; and (iii) one, two, and three dots

Surgical sutures were cut into 50‐cm lengths for tensile testing and 70‐cm lengths for tendon repair. Bidirectional barbs were cut on the size 2–0 and 3–0 PVDF and PP monofilament sutures using a manual barb cutting machine (Figure 2) donated by the former Quill Medical.

Suture clamps were used to hold the suture at both ends and provide tension to keep the suture straight as the barbs were cut. To complete one barbing operation, the following steps were performed: the rubber‐faced compression plate was placed face down on the base; the suture sample was secured in the groove by tightening the positioning screws; the compression plate was removed, and a knife positioning plate (e.g., knife positioning plate I with one dot) was placed on the base; the cutting knives were used to cut the first series of barbs; the cutting machine was rotated 180° without changing the knife positioning plate; the cutting knives were used again to cut the second series of barbs in the opposite direction; the cutting machine was rotated 180° back to its original position; the knife positioning plate I was removed, and the positioning plate II (with same dot number) was placed; and another two series of bidirectional barbs were cut as for the first plate. So, in each barbing operation, knife positioning plates I and II with the same dot number (considered as a pair) were used in order, for a total of four cuts. After each barbing operation, the positioning screws were loosened, the suture clamps were rotated 120°, and the process above was repeated with a different pair of knife positioning plates, selected so that the dot numbers corresponded with those on the suture clamps. A complete fabrication involved three barbing operations, creating bidirectional barbed sutures with barbs staggered around the suture diameter (Figure 2). A video of the barbed suture fabrication process is included in the online Supporting Information.

### 2.3 Barb geometry

**FIGURE 3:**
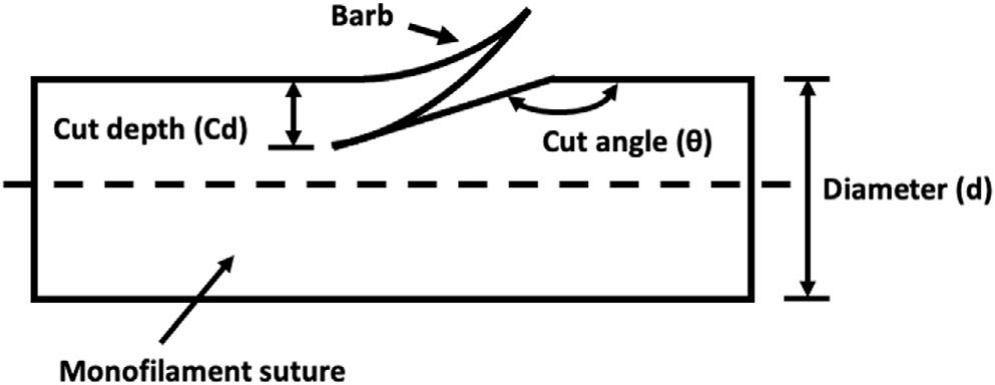
Geometry of a single barb

The geometries of the fabricated barbed sutures (cut depth and cut angle) were determined using a Nikon Eclipse 50i POL optical microscope (Nikon Corp, Tokyo, Japan) at 20x magnification. The number, frequency, angle, and depth of the barbs (Figure 3) were fixed and were determined by the blades on the cutting knife. The nominal values for the barb geometries were 9 barbs within 4 cm with a cut angle of 165° and a cut depth of 20%. Barbed sections were 8 cm in length in each direction with a 2‐mm gap in the middle. After barbing, the sutures were swaged at both ends with diamond‐pointed needles, which are commonly used in tendon repair, and the needles were sized for the corresponding suture sizes (size 22 for size 2–0 suture and size 20 for size 3–0 suture).^27^

### 2.4 Suture tensile test

The breaking force, or ultimate tensile force, is one of the most critical mechanical properties of surgical sutures. The tensile properties of the non‐barbed and barbed monofilament sutures were tested on an Instron 5584 universal testing machine (Instron Corp, Canton, MA) with a 100‐N load cell following the ASTM D3822‐07 “Tensile Properties of Single Textile Fibers” testing guidelines. The suture ends were mounted between flat clamps of the testing machine to set the gauge length at 20 cm. For the barbed sutures, the barbed section was mounted in the middle of the gauge length. After a preload of 2 N was applied, the sutures were tested to failure in uniaxial tension using a crosshead speed of 300 mm/min. Displacement and force data were recorded at 10 Hz and analyzed using Origin software (OriginLab Corp, Northampton, MA). The ultimate tensile force and displacement at failure were measured and recorded at the breaking point. The stiffness was defined as the slope in the initial linear elastic region of the force‐displacement curve. Stiffness indicates the ability of the suture to resist deformation for a given applied tensile force, with greater stiffness providing more resistance to deformation.

### 2.5 Tendon repair

**FIGURE 4:**
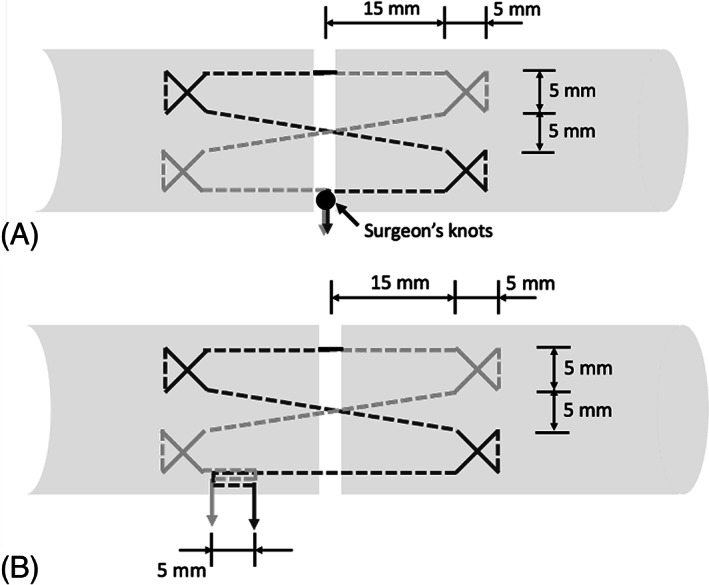
(A) Cross‐locked cruciate suturing technique for traditional non‐barbed sutures; (B) modified cross‐locked cruciate suturing technique for barbed sutures

The tendons were thawed at room temperature for 24 h before surgical repairs were performed. All repairs were performed by a single operator (YH) using either a 4‐strand cross‐locked cruciate suturing technique for the non‐barbed surgical sutures or a 4‐strand modified cross‐locked cruciate suturing technique for the barbed surgical sutures (Figure 4). Each suture was used to repair one porcine patellar tendon.

The starting points for the suturing techniques are indicated as the black solid line in the middle of the top strand in Figure 4, with dark and light grey lines representing the two insertion directions; for barbed sutures, they also represent the two directions of the barbs. For the repair with the barbed sutures, the 2‐mm long non‐barbed gap was placed in the center of the repair site, and the first 2 cross‐locked anchors were made on the two sides of the repaired tendon, with the barbs pointing in one direction, and the other 2 cross‐locked anchors were made using the barbed suture with the barbs pointing in the opposite direction. After making all 4 cross‐locked anchors, the barbed sutures were locked at a point 10 mm away from the repair site. For the traditional non‐barbed sutures, the starting point was at the center of the tendon transection site, and a 5‐mm wide and 15‐mm long stitch was inserted before making a cross‐locked anchor on each side of the transection site. After making 4 symmetric cross‐locked anchors, the monofilament suture was then tied at the insertion site using a surgeon's knot.

### 2.6 Tendon suture pullout test

To examine the anchoring performance of the barbed suture within the surrounding tissue, the maximum force during a tendon suture pullout test was calculated as an indicator for how much force the repaired tissue could support during early active motion, such as post‐surgical physical rehabilitation. The tendons were mounted between flat, stainless steel clamps and, after applying a preload of 2 N, were tested to failure in uniaxial tension using a crosshead speed of 20 mm/min (Instron 5584 mounted with a 2000‐N load cell). The maximum force was recorded.

Gap formation was also monitored as a measure of the surgical efficacy of the repair and the anchoring strength of the suture material. Clinically, if the gap at the repair site widens more than 2 mm, then tendon function is lost, and the repair is considered a failure.^17^, ^28^ To monitor the gap size formed at the repair site during the pullout test, a dial caliper set to a 2‐mm gap was placed adjacent to the tendon. Gap formation was recorded with a high‐definition video camera (Canon VIXIA HF R62, Canon Inc., Tokyo, Japan) that was manually synchronized with the load cell output. After testing, the video images were analyzed frame‐by‐frame using ImageJ software (National Institutes of Health, Bethesda, MD) to determine the force required to generate a 2‐mm gap, which was defined as failure of the tendon repair.

### 2.7 Statistical analysis

Suture morphology and mechanical testing data were compared across suture material (PVDF, PP) and suture type (2–0 non‐barbed, 2–0 barbed, 3–0 non‐barbed) using two‐way ANOVA with Tukey's adjustments for multiple comparisons and a significance level of 0.05 (JMP Pro13, SAS Institute, Cary, NC). Data are reported as mean ± *SD*.

## 3 RESULTS

### 3.1 Barb geometry

**FIGURE 5:**
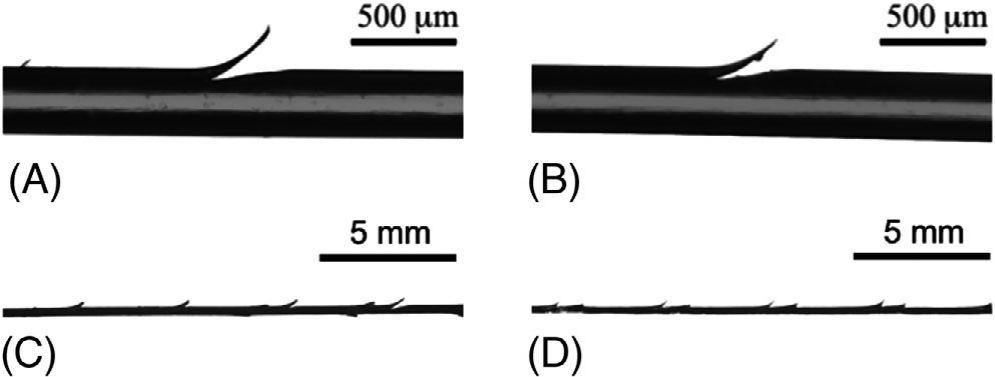
Representative microscopic images of a single barb on the surface of 2–0 barbed sutures made with (A) PVDF and (B) PP materials. The cutting process produced barbs with greater bending from the suture surface for PVDF compared to PP sutures. Lower magnification images show several barbs on the surface of the (C) PVDF and (D) PP sutures, which are separated by 120° rotation about the suture long axis

Although the barb geometry was set at a nominal cut angle of 165° and a nominal cut depth of 20%, the actual measured values varied a small amount (Table 1). The microscopic barb geometries on the PVDF and PP sutures showed different shapes, reflecting the different degrees of barb bending resulting from the cutting process, with the barbs on the PVDF sutures bending away from the surface more than the barbs on the PP sutures (Figure 5). More bending may cause the PVDF suture to bend or peel differently than the PP suture when inserted into surrounding tissue and loaded with an applied force.

**TABLE 1:** Measured barb geometries (mean ± *SD*), *n =* 5 per group

Group	Cut angle (°)	Cut depth (%)
PVDF 2–0 Barbed	167.2 ± 3.1	21.1 ± 3.1
PP 2–0 Barbed	165.9 ± 1.7	22.4 ± 2.4

### 3.2 Suture tensile test

**FIGURE 6:**
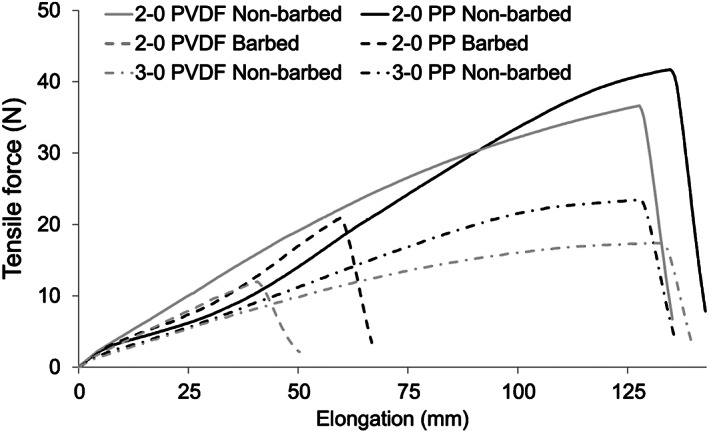
Representative tensile force‐elongation curves for PVDF and PP monofilament nonbarbed and barbed sutures

**FIGURE 7:**
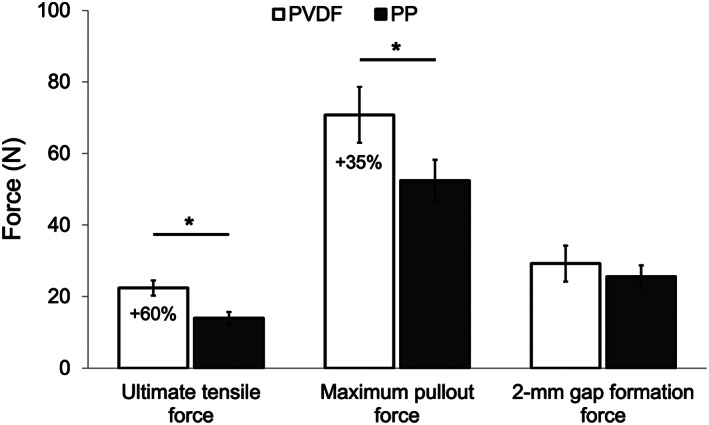
Comparison of the ultimate tensile force, maximum pullout force, and 2‐mm gap formation force of PVDF and PP 2–0 barbed sutures. *p < .05

The ultimate tensile force decreased significantly after cutting the barbs (Figure 6, Table 2), with values that were 40.9% lower for the PVDF size 2–0 sutures (*p <* .001) and 59.2% lower for the PP size 2–0 sutures (*p <* .001). The ultimate tensile force was significantly higher in PVDF sutures than in PP sutures for 2–0 non‐barbed sutures (10.5% greater, *p <* .001), 2–0 barbed sutures (60.0% greater, *p <* .001, Figure 7), and 3–0 non‐barbed sutures (23.1% greater, *p <* .001). The ultimate tensile force of PVDF 2–0 barbed sutures was similar to that of PVDF 3–0 non‐barbed sutures (*p =* 0.90), consistent with the information provided by the manufacturers stating that the tensile properties of a barbed suture are equivalent to the properties of a non‐barbed suture of the same material that is one size smaller.^26^ However, different from PVDF, PP 2–0 barbed sutures required significantly less peak force to break than did PP 3–0 non‐barbed sutures (*p <* .001).

**TABLE 2:** Suture tensile test and tendon suture pullout test results (mean ± *SD*), *n =* 8 per suture material/size/type

Suture material	Suture size/type	Ultimate tensile force (N)	Stiffness (N/m)	Maximum pullout force (N)	2‐mm gap formation force (N)
PVDF	2–0 NB	37.9 ± 1.3^a^ ^,^ ^b^ ^,^ ^c^	360.1 ± 10.6^a^ ^,^ ^c^	104.9 ± 9.5^b^ ^,^ ^c^	24.5 ± 6.2^a^ ^,^ ^c^
	2–0 B	22.4 ± 2.1^a^	308.0 ± 30.5^d^	70.8 ± 7.8^a^ ^,^ ^d^	29.2 ± 5.0^d^
	3–0 NB	22.5 ± 0.5^a^	226.2 ± 6.7^a^	59.1 ± 10.2	14.4 ± 2.7^a^
PP	2–0 NB	34.3 ± 1.4^b^ ^,^ ^c^	326.8 ± 13.9^c^	103.2 ± 14.9^b^ ^,^ ^c^	19.3 ± 2.6^b^
	2–0 B	14.0 ± 1.7^d^	351.7 ± 49.7^d^	52.4 ± 5.8^d^	25.6 ± 3.1^d^
	3–0 NB	18.2 ± 1.1	174.9 ± 14.0	65.1 ± 4.6	18.3 ± 3.6

Abbreviations: B, barbed; NB, non‐barbed; PP, polypropylene; PVDF, polyvinylidene fluoride.

*Note*: *p <* .05.

^a^ PVDF versus PP of same size and type.

^b^ 2–0 NB versus 2–0 B of same material.

^c^ 2–0 NB versus 3–0 NB of same material.

^d^ 2–0 B versus 3–0 NB of same material.

Stiffness was lower for size 3–0 than size 2–0 sutures (Table 2). For PVDF, the 3–0 suture stiffness was 37.2% lower than that for 2–0 non‐barbed sutures (*p <* .001) and 26.6% lower than that for 2–0 barbed sutures (*p <* .001). Similarly, for PP, 3–0 suture stiffness was 46.5% lower than for 2–0 non‐barbed sutures (*p <* .001) and 50.3% lower than for 2–0 barbed sutures (*p <* .001). Although PVDF sutures were stiffer than PP sutures for size 2–0 non‐barbed sutures (*p <* .001) and size 3–0 non‐barbed sutures (*p <* .001), PVDF and PP suture stiffness was more similar for size 2–0 barbed sutures (*p =* .052).

### 3.3 Tendon suture pullout test

The maximum force during the tendon suture pullout test represented the force required either to break the suture or pull it out of the tendon tissue. Consistent with the suture ultimate tensile force, the maximum pullout test force was greatest for the 2–0 non‐barbed sutures (Table 2). Compared to the PVDF 2–0 non‐barbed sutures, maximum pullout force was 32.5% lower for the PVDF 2–0 barbed sutures (*p <* .001) and 43.7% lower for the PVDF 3–0 non‐barbed sutures (*p <* .001). Similarly, compared to PP 2–0 non‐barbed sutures, maximum pullout force was 49.2% lower for PP 2–0 barbed sutures (*p <* .001) and 36.9% lower for PP 3–0 non‐barbed sutures (*p <* .001). Pullout force for PVDF 2–0 barbed suture was 35.1% greater than for PP 2–0 barbed sutures (*p <* .001, Figure 7) and 19.8% greater than for PVDF 3–0 non‐barbed sutures (*p =* .022). The maximum pullout force did not differ significantly between the PVDF and PP materials for either 2–0 non‐barbed or 3–0 non‐barbed sutures. These results show that a reduction in suture cross‐sectional area, either by cutting barbs along the surface or selecting a smaller size, more negatively affects the performance of the tendon repair than does the suture material.

The force required to form a 2‐mm gap, which was defined as failure for the tendon repair, was 32.6% larger for the PP 2–0 barbed sutures than for the PP non‐barbed sutures of the same size (*p <* .001, Table 2). The 2‐mm‐gap force of PVDF 2–0 barbed sutures was similar to that of PVDF 2–0 non‐barbed sutures (*p =* .12). In addition, the 2‐mm‐gap force was equivalent between the PVDF and PP materials for the 2–0 barbed sutures (*p =* .11, Figure 7) but was marginally greater for PVDF than for PP in 2–0 non‐barbed sutures (*p =* .046) and marginally lower for PVDF than for PP in 3–0 non‐barbed sutures (*p =* .028). Also, significantly more force was required to form the 2‐mm gap in 2–0 barbed sutures than in 3–0 non‐barbed sutures, both for PVDF (*p <* .001) and PP (*p <* .001) materials.

## 4 DISCUSSION

Although barbed sutures have been available for surgeons to use for several decades, the US FDA has only approved the use of biodegradable barbed sutures for plastic and cosmetic surgeries (e.g., rhytidectomy and breast reconstruction) and for soft tissue endoscopic surgeries (e.g., laparoscopic and urologic procedures) but not yet for tendon repair. Because the commonly used suture material PP is associated with the problems of thrombogenicity, creep, and long‐term mechanical fatigue, PVDF is considered an attractive alternative suture material due to less thrombogenicity and superior long‐term mechanical fatigue performance. In this study, we successfully fabricated PVDF barbed sutures using a manual barb cutting machine. These novel PVDF barbed sutures showed superior mechanical properties (8.4 N greater ultimate tensile strength for size 2–0 sutures) and anchoring performance (18.4 N greater tendon suture pullout strength) compared with PP barbed sutures when used for tendon repair. A sufficiently strong tendon repair is needed so that the tendon can tolerate the forces generated during early active motion in rehabilitation programs. Previous studies have reported that tenomalacia at the suture‐tendon junction site caused 50% loss of initial strength of the immobilized sutures within the first week, whereas the breaking force for the tendon repair depends on the mechanical properties of the sutures, as well as the local anchoring performance of the suture‐tissue interface.^29^, ^30^, ^31^

Our study demonstrated that PVDF non‐barbed sutures had a higher ultimate tensile force than PP non‐barbed sutures of the same size. The greater tensile properties of PVDF non‐barbed sutures may be related to the higher crystallinity of PVDF (59%) compared to PP (43%), since with higher crystallinity, the intermolecular bonding is more significant, which would lead to increased strength.^32^ Previous studies have also reported superior properties for PVDF sutures compared with PP sutures. With 9 years exposure to hydrolytic conditions, PVDF sutures lost less of their initial tensile strength (7.5%) compared to PP sutures (46.6%).^8^ One previous study found no significant difference between 5–0 PVDF and PP sutures in terms of tensile strength (9.2 N for both) or percent elongation (40.0% for both) but reported that PVDF sutures had significantly higher knot pull strength (6.7 N) than PP sutures (5.9 N); and, despite greater initial extensibility during creep testing (30.5% at 30 min compared to 21.0% for PP), PVDF sutures had more long‐term creep resistance with only 5.1% additional stretching in the next 11.5 h compared to 15.0% for PP sutures.^22^ Another study also found similar greater extensibility and resistance to creep of PVDF sutures compared to PP sutures of the same size, suggesting PVDF sutures will have more long‐term dimensional stability than PP sutures. In addition, in an in vivo canine thoracoabdominal bypass model, PVDF sutures did not show evidence of surface stress cracking after 2 years that was visible on PP sutures.^9^, ^20^ The absence of surface cracks and fibrillation that has also been reported in these localized damage areas makes PVDF sutures less susceptible than PP sutures to chemical degradation in vivo, which suggests that PVDF sutures will have better long‐term performance and fatigue resistance.^22^ When used for flexor tendon repair, PVDF sutures had significantly higher maximum pullout force (31.9 vs. 26.6 N) and 2‐mm‐gap formation force (22.7 vs. 19.9 N) compared with PP sutures.^22^

The barbs effectively anchored the tendon tissue along the whole length of the suture, preventing suture slipping or pull out with forces applied in the direction opposite to suture insertion. Because the barbed sutures in this study were bidirectional, they were resistant to applied loads in either direction. Although fabrication of the barbs decreased the ultimate tensile force of both suture materials, the PVDF barbed sutures were less affected and had a substantially higher breaking force compared to the PP barbed sutures. This finding confirmed our hypothesis that PVDF barbed sutures provide an improved mechanical performance compared to PP barbed sutures. The barbs on the PVDF and PP barbed sutures had different shapes, reflecting the different degrees of barb bending; as evident in Figure 5, the barbs on the PP sutures were bent backward more than the barbs on the PVDF sutures, likely due at least in part to the lower stiffness of PP (360.1 N/m) compared to PVDF (326.8 N/m).

Assessing adequacy of suture properties, a previous study reported that the force in the human flexor digitorum profundus and flexor digitorum superficialis tendons ranged 2–9 N during active extension and passive flexion and 2–19 N during active unresisted flexion, indicating that the tensile strength of the 2–0 barbed PVDF sutures (22.4 N) is more appropriate than the tensile strength of 2–0 barbed PP sutures (14.0 N).^33^ A previous study of only PP 2–0 barbed sutures reported an ultimate tensile force of 40 N, which was higher than the 14 N in our study.^34^ This discrepancy can likely be explained by different barb cutting parameters, as our previous work showed that the peak tensile force decreased from 33 to 26 N when the cut depth increased from 0.07 to 0.18 mm with a constant cut angle of 160°.^4^, ^20^

The strength of a tendon repair is governed by several factors, including, but not limited to, the configuration or suture pattern, number of strands crossing the repair, tensile strength of the suture, and suture pullout force, an indicator of the suture anchoring performance. In this study, we used a cruciate repair, which is one of the most commonly used suturing techniques in tenorrhaphy, and various researchers have shown that the cross‐lock cruciate configuration is biomechanically the most suitable choice for tendon repair, since it optimizes the gap formation force and the maximum force to failure.^21^, ^25^ With this technique, we showed a greater tendon suture pullout force for the PVDF size 2–0 barbed sutures (71 N) compared to the PP 2–0 barbed sutures (52 N) and the PVDF 3–0 non‐barbed sutures (59 N), supporting our hypothesis for better anchoring performance with PVDF barbed sutures. Another study using other materials and repair techniques for a human cadaver torn flexor tendon showed a maximum pullout force of 35 N when using a size 3–0 Ethibond suture with a Kessler repair and 30 N when using a size 2–0 barbed Quill suture with a Kessler–Bunnell repair, both of which were 2‐strand repairs.^22^ Considering the number of sutures passing through the repair site was different in that study (twice compared to four times in ours), our study demonstrated a comparative barb anchoring performance in terms of force per pass but superior suturing efficacy by increasing the number of sutures passed through the repair site with the 4‐strand cross‐locked cruciate suturing technique.

Multiple suture strands crossing the repair site are needed to allow early postoperative movements without damaging the tenorrhaphy, and currently 4‐strand repairs are the minimum recommendation for tendon repairs.^24^ A previous study of flexor tendon repair in human cadavers suggested that the number of core suture strands across the repair site played a more important role in the strength of tendon repair than did suture size; they found that an 8‐strand repair using 4–0 sutures was 43% stronger than a 4‐strand repair using 3–0 sutures, even though the 3–0 suture was 49% stronger than the 4–0 suture made from the same material.^17^ In other studies using a 4‐strand repair, locking cross‐stitches had significant benefits compared to looped techniques with regard to greater gap formation force.^7^, ^24^, ^25^

One limitation of this study was that, while the barb cutting machine provided the desired geometry and uniformity of barbs, the procedure was performed manually, since PVDF barbed sutures are not yet commercially available. More studies are needed to evaluate the efficacy of commercial barbed sutures, which are created on a continuous production machine, in tendon repair. In addition, the repairs were performed by a researcher under the advisement of an experienced orthopedic surgeon, rather than directly by the surgeon, which likely introduced more variable results. Nevertheless, this study showed that although the mean 2‐mm‐gap formation force did not differ significantly between the PVDF and PP 2–0 barbed sutures, the combined greater degree of bending in PVDF barbs and superior tendon pullout force indicate that the PVDF barbed sutures show promise for use in tendon repair.

## 5 CONCLUSIONS

This study showed that barbed sutures can be fabricated with PVDF monofilament sutures and have the potential to be used for tendon repair. The PVDF knotless barbed sutures showed superior mechanical properties, with higher ultimate tensile strength and stiffness and higher maximum tendon pullout force, compared with PP barbed sutures. They also showed excellent anchoring performance, with higher 2‐mm‐gap formation force than non‐barbed sutures of the same size. Future work will focus on improving the efficiency and reliability of the barb cutting process, as well as performing cyclic tests that simulate long‐term in vivo loading to assess the clinical advantage of using PVDF barbed sutures over traditional knotted PP and PVDF sutures.

## Supporting information


Video S1

## Data Availability

The data that support the findings of this study are available from the corresponding author upon reasonable request.
